# Sirtuins in mitophagy: key gatekeepers of mitochondrial quality

**DOI:** 10.1007/s11010-025-05358-0

**Published:** 2025-07-24

**Authors:** Francisco Alejandro Lagunas-Rangel

**Affiliations:** 1 Laboratory of Pharmaceutical Pharmacology, Latvian Institute of Organic Synthesis, Aizkraukles 21, Riga, 1006 Latvia; 2 Department of Surgical Sciences, Uppsala University, BMC Box 593, Husargatan 3 Uppsala, 75124 Sweden

**Keywords:** Mitochondria, Receptor-mediated mitophagy, Ubiquitin-mediated mitophagy, PINK1-PARKIN pathway, FOXO transcription factors

## Abstract

Mitochondria are highly dynamic organelles essential for cellular energy production. However, they are also a primary source of reactive oxygen species, making them particularly vulnerable to oxidative damage. To preserve mitochondrial integrity, cells employ quality control mechanisms such as mitophagy, a selective form of autophagy that targets damaged or dysfunctional mitochondria for degradation. Among the key regulators of mitophagy are the sirtuins, a family of NAD^+^-dependent deacetylases. SIRT1, SIRT3, and SIRT6 generally promote mitophagy, whereas SIRT2, SIRT4, SIRT5, and SIRT7 often act as negative regulators. Sirtuin-mediated regulation of mitophagy is critical for maintaining cellular homeostasis and is implicated in a variety of physiological and pathological conditions. The aim of this review is to provide an overview focused on describing how sirtuins influence the mitophagy process. It highlights the different molecular mechanisms by which individual members of the sirtuin family modulate mitophagy, either by promoting or suppressing it, depending on the context. In addition, the review explores the relevance of sirtuin-regulated mitophagy in health and disease, emphasizing some conditions under which altered sirtuin activity could be harnessed for therapeutic benefit.

## Introduction

Mitochondria are dynamic intracellular organelles often described as the “powerhouse of the cell” because of their central role in the production of adenosine triphosphate (ATP), the cell’s main energy currency [1]. However, their functions go far beyond energy production. Mitochondria are involved in a wide range of cellular processes, including phospholipid biosynthesis and transport, calcium signaling, iron metabolism, and apoptosis, among many others [2].

Mitochondria are composed of two distinct membranes: the outer membrane and the inner membrane, which together define two internal compartments: the intermembrane space (IMS) and the matrix. The inner membrane is extensively folded into structures called cristae, which increase the surface area available for critical metabolic reactions [3]. The matrix contains the enzymes of the tricarboxylic acid (TCA) cycle, which is essential for the oxidative breakdown of carbohydrates and fatty acids. In this cycle, acetyl-CoA is oxidized to carbon dioxide (CO_2_), a process coupled to the reduction of NAD^+^ and FAD to NADH and FADH_2_ [4]. These reduced cofactors transfer high-energy electrons to the electron transport chain, located in the inner membrane. As electrons are transferred through the chain to molecular oxygen, the energy released is used to pump protons across the inner membrane, creating an electrochemical gradient. This proton gradient drives ATP synthesis through oxidative phosphorylation, the main energy-producing process in aerobic cells [5].

Mitochondria are a major source of reactive oxygen species (ROS), which are generated as by-products of the electron transport chain. This makes them particularly susceptible to oxidative stress, which can lead to structural and functional damage [6]. Beyond oxidative stress, factors such as calcium overload and mitochondrial DNA mutations can also alter mitochondrial integrity, impairing its function and contributing to overall cellular dysfunction [7, 8]. To maintain mitochondrial integrity, cells rely on several quality control mechanisms, such as ROS scavenging, DNA repair, refolding, or degradation of misfolded proteins and mitochondrial fusion and fission [9]. A key component of this system is mitophagy, a selective form of autophagy that removes damaged or dysfunctional mitochondria [10]. During mitophagy, damaged mitochondria are encapsulated and sent to lysosomes for degradation and recycling [2]. This process is essential for maintaining mitochondrial quality and the proper number of mitochondria, thus contributing to overall cellular homeostasis [11].

On the other hand, the sirtuin family comprises a group of structurally and functionally highly conserved proteins found in all domains of life, including eubacteria, archaea, and eukaryotes [12, 13]. In mammals, there are seven sirtuin isoforms (SIRT1-SIRT7), each of which plays critical roles in the regulation of diverse cellular processes such as cell growth, energy metabolism, stress response, inflammation, circadian rhythms, neuronal function, and aging [14, 15]. Each sirtuin isoform has a particular subcellular localization, with SIRT3, SIRT4, and SIRT5 primarily in the mitochondria, SIRT6 and SIRT7 in the nucleus, and SIRT1 and SIRT2 found in both the nucleus and cytoplasm [16–18]. Although sirtuins are known primarily as NAD^+^-dependent deacetylases, they also catalyze other reactions, such as ADP-ribosylation, desuccinylation, demalonylation, delactylation, debutyrylation, decrotonylation and defatty-acylation [19]. Notably, their dependence on NAD^+^ as a coenzyme functionally links sirtuin activity to cellular energy status and nutrient availability [20].

With this context in mind, the purpose of this review is to present a concise overview of the latest and most significant discoveries connecting sirtuins to the regulation of mitophagy. It focuses on the specific molecular mechanisms through which individual sirtuins coordinate this process. Furthermore, the review examines both physiological and pathological conditions where sirtuin-driven mitophagy plays a significant role, highlighting scenarios in which modulation of sirtuin activity, either to promote or inhibit mitophagy, could offer therapeutic benefits.

## Mammalian mitophagy

**Fig. 1:**
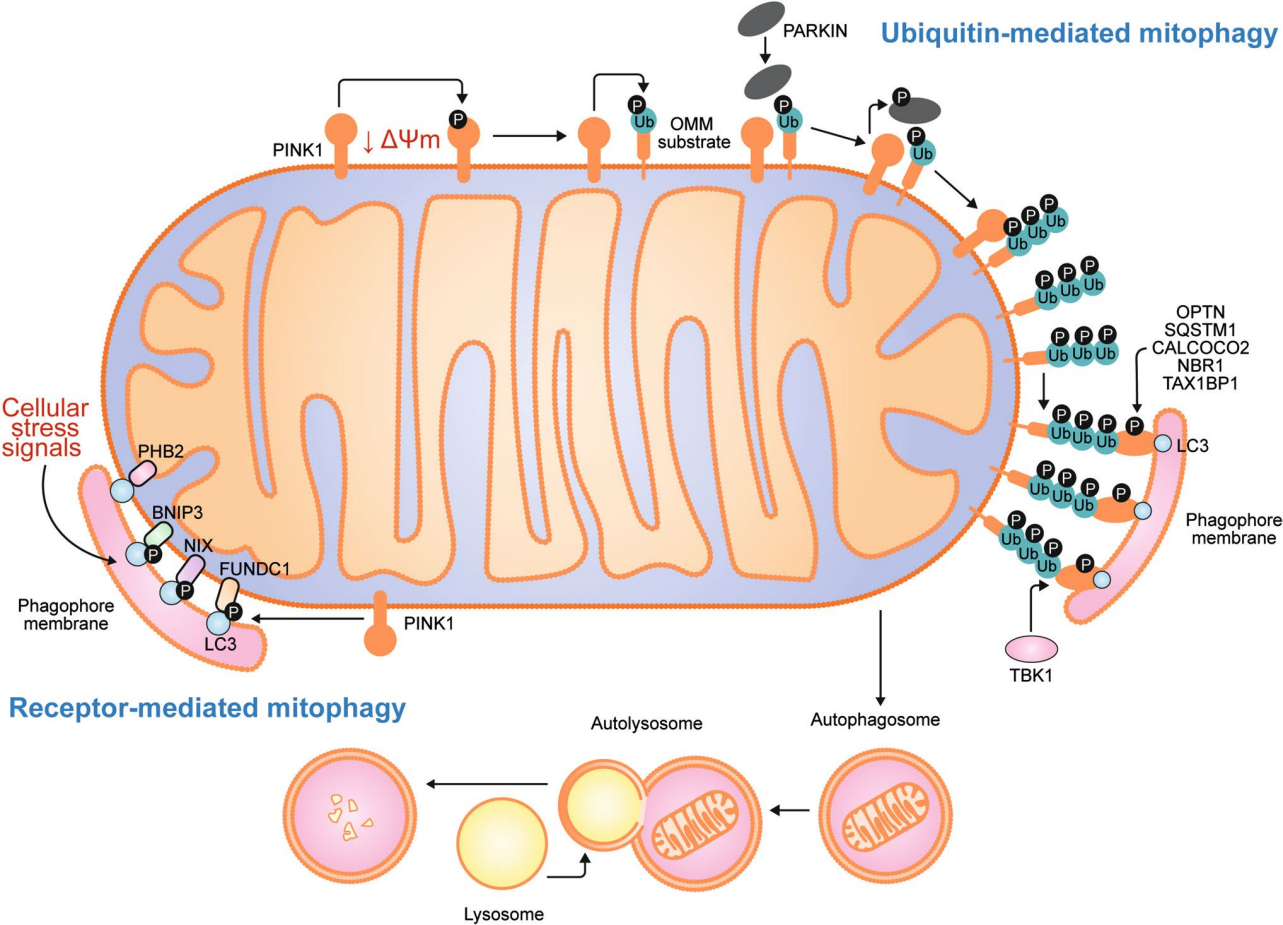
Mitophagy mechanisms**.** Mitophagy can proceed through both ubiquitin-dependent and receptor-mediated pathways. In ubiquitin-mediated mitophagy, mitochondrial damage and loss of membrane potential (ΔΨm) stabilize PINK1 on the outer mitochondrial membrane (OMM), where it recruits and activates PARKIN. PARKIN ubiquitinates multiple OMM proteins, forming polyubiquitin (poly-Ub) chains that are phosphorylated by PINK1, serving as “eat me” signals for the autophagy machinery. Adaptor proteins such as OPTN, SQSTM1/p62, CALCOCO2/NDP52, NBR1, and TAX1BP1 recognize these phosphorylated poly-Ub chains and link damaged mitochondria to autophagosomes via interaction with LC3. TBK1 further enhances this process by phosphorylating adaptor proteins, increasing their affinity for ubiquitinated substrates. In receptor-mediated mitophagy, proteins such as BNIP3, NIX, and FUNDC1 are embedded in the OMM and directly interact with LC3 to drive mitophagosome formation. PHB2, normally located in the inner mitochondrial membrane, is exposed to the OMM upon mitochondrial damage and also binds LC3. The expression and activation of specific receptors vary across tissues and stimuli, adding specificity to the process. Phosphorylation of BNIP3 and NIX enhances their LC3-binding ability, and these receptors also contribute to mitochondrial fission by promoting OPA1 disassembly and recruiting DRP1. Notably, PARKIN-mediated ubiquitination of NIX and BNIP3 illustrates the functional crosstalk between receptor-driven and ubiquitin-mediated mitophagy pathways

Mitophagy is promoted through mechanisms mediated by ubiquitin or by specific receptors located on the mitochondrial outer membrane, both of which lead to the formation of autophagosomes that engulf and degrade damaged mitochondria. Receptor-mediated mitophagy is triggered by various cellular stress signals (such as hypoxia or energy deprivation), whereas ubiquitin-mediated mitophagy is usually activated by a loss of mitochondrial membrane potential (Fig. 1). Notably, although these two mechanisms may operate independently, they often intersect and influence each other [2].

In healthy mitochondria, PTEN-induced putative kinase 1 (PINK1) is rapidly degraded through voltage-dependent proteolysis, keeping its levels low under normal conditions [21]. In ubiquitin-mediated mitophagy, mitochondrial damage triggers a loss of membrane potential (depolarization), leading to the stabilization and accumulation of PINK1 dimers at the outer mitochondrial membrane [22]. These dimers then undergo autophosphorylation at PINK1 serine residues S228 and S402, and subsequently phosphorylates the E3 ubiquitin ligase PARKIN at S65 residue, leading to its activation [23]. Activated PARKIN promotes polyubiquitination of several outer mitochondrial membrane proteins, which in turn recruits mitophagy adaptors such as optineurin (OPTN), sequestosome-1 (SQSTM1 or p62), calcium-binding and coiled-coil domain-containing protein 2 (CALCOCO2 or NDP52), next to BRCA1 gene 1 protein (NBR1), and tax1-binding protein 1 (TAX1BP1) [24, 25]. These adaptors contain microtubule-associated protein 1 light chain 3γ (MAP1LC3C or LC3)-interacting regions (LIRs) that allow them to bind to LC3 on the forming autophagosome (phagophore), thereby initiating the engulfment of damaged mitochondria [26].

Adaptor function is regulated by TANK-binding kinase 1 (TBK1), which phosphorylates them to increase their binding affinity for ubiquitin chains and autophagy-related protein 8 (ATG8) family members [27]. Additionally, CALCOCO2 and OPTN play roles in recruiting early autophagy components. CALCOCO2 facilitates the recruitment of the unc-51-like kinase 1 (ULK1) complex through RB1-inducible coiled-coil protein 1 (RB1CC1), while OPTN helps deliver autophagy-related protein 9 (ATG9)-positive vesicles to sites of autophagosome formation [28].

The elongation of the phagophore membrane around mitochondria is a selective process that involves the TBC1 domain family members TBC1D15 and TBC1D17 [29]. These proteins contain LIRs and function as GTPase-activating proteins (GAPs) for RAB-type GTPases, which regulate vesicular membrane fusion. Mitochondrial fission protein 1 (FIS1) directs them to the outer mitochondrial membrane [30]. Additionally, RAB5 GDP/GTP exchange factor (RABGEF1), an upstream regulator of the RAB GTPase cascade, is recruited to ubiquitinated mitochondria downstream of PARKIN. RABGEF1 facilitates the recruitment and activation of RAB5 and RAB7, which coordinate endosomal trafficking essential for mitophagy progression [31]. The RAB cycle assembles ATG9 vesicles and extends the isolation membrane to envelop the mitochondria.

The final stage of mitophagy (autophagosome–lysosome fusion) is mediated by the ATG8 protein family, which includes LC3 (LC3A, LC3B, LC3C) and γ-aminobutyric acid receptor-associated proteins (GABARAP, GABARAP-L1, GABARAP-L2) [32]. These proteins are conjugated to phosphatidylethanolamine through two ubiquitin-like conjugation systems, allowing them to associate with both the expanding phagophore and mature autophagosomes [33]. A pivotal step in autophagosome-lysosome fusion is membrane fusion mediated by the soluble N-ethylmaleimide-sensitive factor attachment protein receptor (SNARE) complex. This process is mainly facilitated by the autophagosome-associated SNAREs syntaxin-17 (STX17) and synaptosomal-associated protein 29 (SNAP29), together with lysosome-associated vesicle-associated membrane protein 8 (VAMP8) or VAMP7 [34].

In the case of receptor-mediated mitophagy, mitochondrial membrane proteins with LIR domains can bind directly to LC3 in the phagophore, bypassing the need for ubiquitination. These receptors include FUN14 domain-containing protein 1 (FUNDC1), BCL-2/adenovirus E1B 19 kDa-interacting protein 3 (BNIP3) and BNIP3-like protein 3 (BNIP3L or NIX), all of which are localized to the outer mitochondrial membrane. Prohibitin-2 (PHB2), located in the inner mitochondrial membrane, also functions as a mitophagy receptor [2]. Interestingly, BNIP3 and NIX can potentiate PINK1/PARKIN-mediated mitophagy through multiple mechanisms. NIX is ubiquitinated by PARKIN, facilitating recruitment of the mitophagy adaptor NBR1 and promoting autophagosome formation [35]. In addition, BNIP3 interacts with PINK1 to promote its accumulation in the outer mitochondrial membrane, thereby promoting PARKIN translocation to damaged mitochondria [36].

## Sirtuin regulation mechanisms on mitophagy

**Fig. 2:**
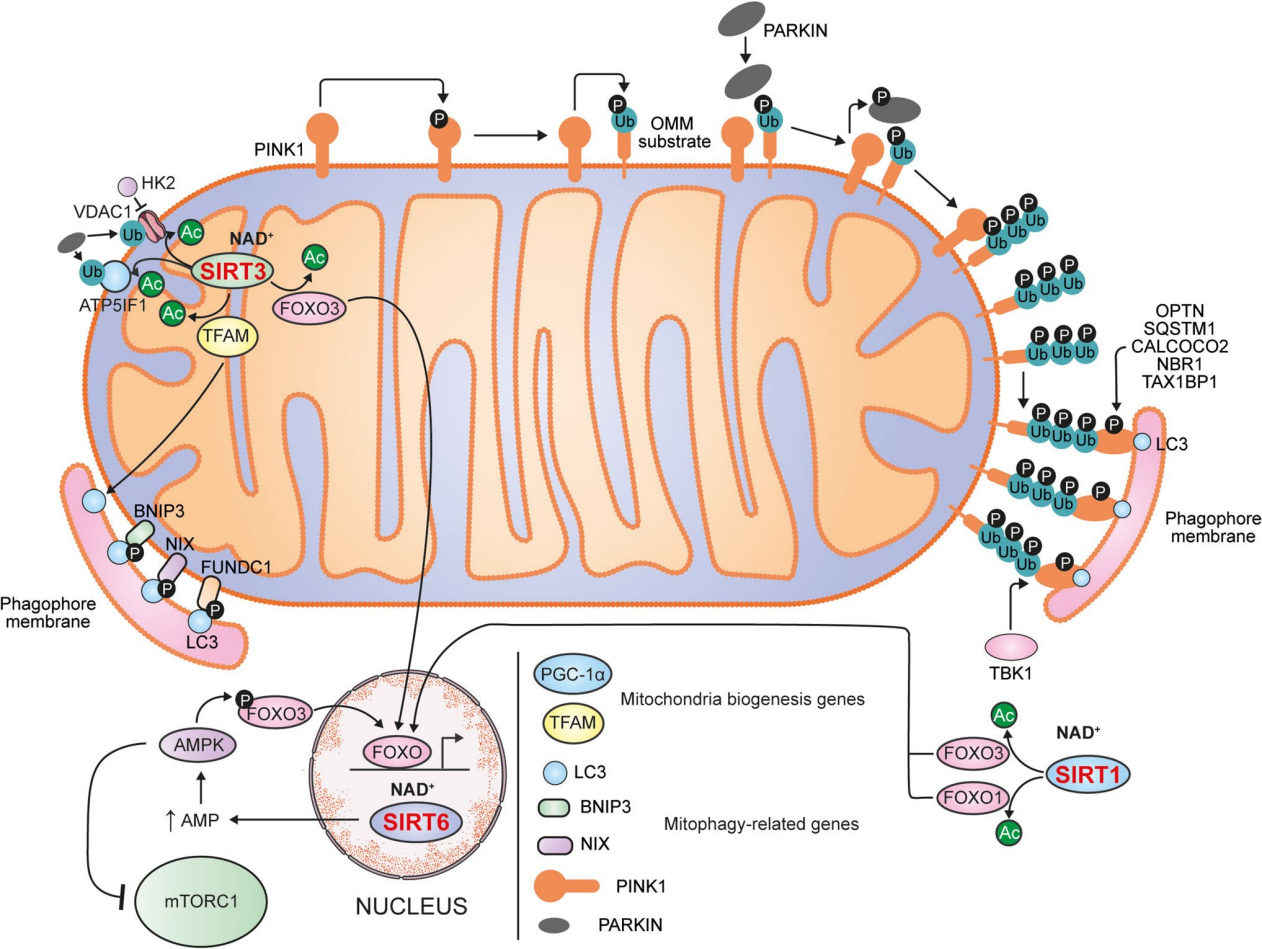
Sirtuins that positively regulate mitophagy. SIRT1 promotes mitophagy by activating the transcription factors FOXO1 and FOXO3, which induce the expression of genes involved in mitochondrial degradation. SIRT3 also enhances mitophagy through activation of FOXO3 and further contributes by deacetylating TFAM which interacts with LC3 to facilitate mitophagosome formation. Additionally, SIRT3 supports PARKIN recruitment by promoting HK2 dissociation from VDAC1 and deacetylating ATP5IF1. SIRT6 contributes indirectly by activating AMPK, which in turn stimulates FOXO3 activity

**Fig. 3:**
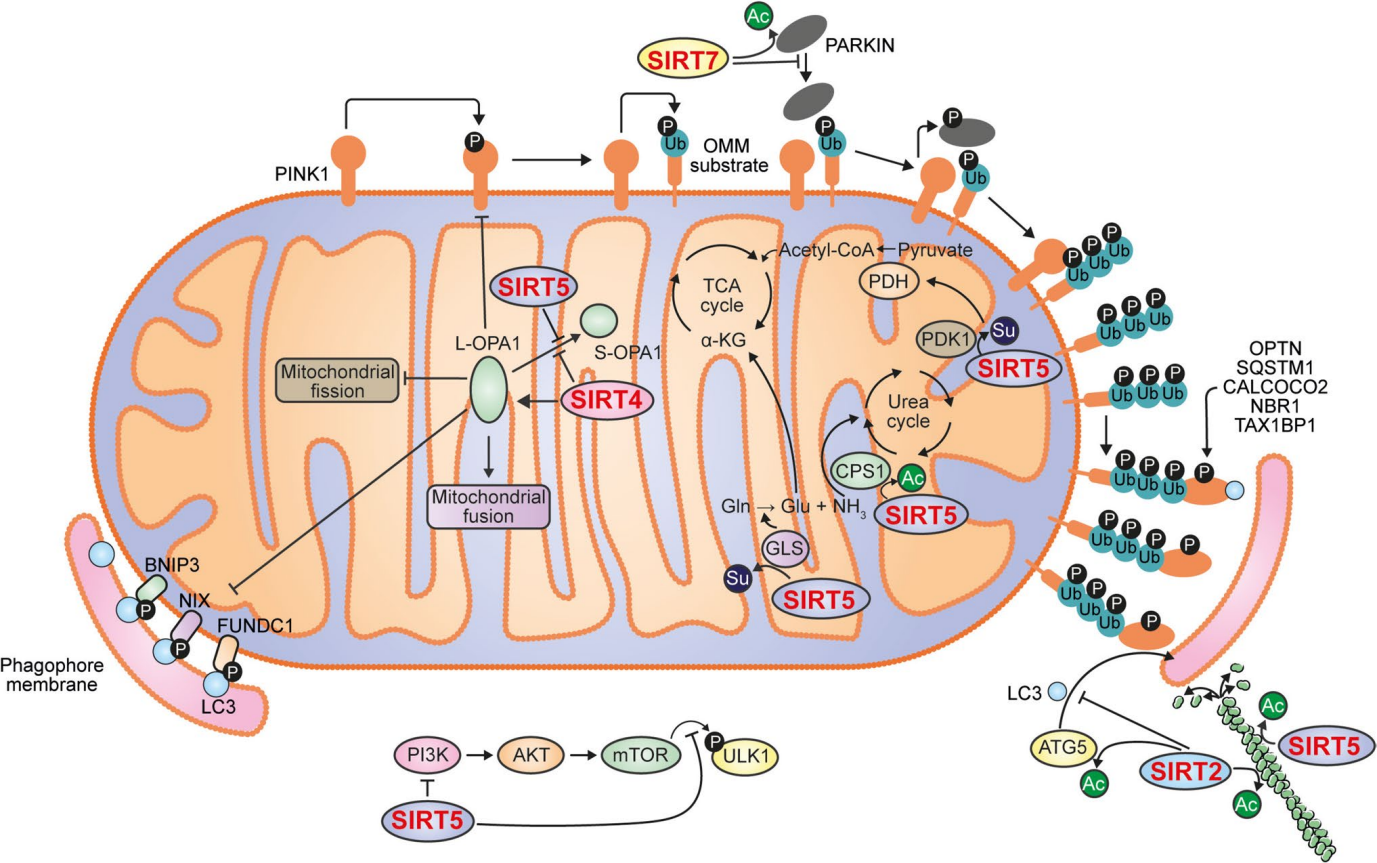
Sirtuins that negatively regulate mitophagy. SIRT2 impairs mitophagy by destabilizing microtubules through the deacetylation of tubulin and by deacetylating ATG5, which hinders the conjugation of LC3B to phosphatidylethanolamine, a key step in autophagosome formation. SIRT4 inhibits mitophagy by binding to and stabilizing the long form of OPA1. SIRT5 exerts multiple inhibitory effects: it deacetylates and activates CPS1 for ammonia detoxification via the urea cycle, and desuccinylates GLS, enhancing the conversion of glutamine (Gln) to glutamate (Glu). It also inhibits ULK1 activity through suppression of the PI3K/AKT signaling pathway. Additionally, SIRT5 desuccinylates and inhibits PDK1, which enhances PDH activity and reduces the cellular reliance on mitophagy. Like SIRT2, SIRT5 also regulates cytoskeletal dynamics by deacetylating β-tubulin. Finally, SIRT7 suppresses mitophagy by maintaining PARKIN in a deacetylated state, thereby preventing its activation and mitochondrial recruitment

The seven human sirtuins contribute to the regulation of mitophagy through distinct molecular mechanisms. The cellular context and the type of stimulus determine the specific sirtuin and signaling pathway involved. Although several pathways can be activated at the same time, their activation is usually tailored to the specific needs of the cell. Figure 2 schematizes the mechanisms by which sirtuins that positively regulate mitophagy exert their effects, while Fig. 3 illustrates the mechanisms by which sirtuins that negatively regulate mitophagy work.

SIRT1 promotes mitophagy by deacetylating and stabilizing the transcription factors forkhead box protein O1 (FOXO1) and/or forkhead box protein O3 (FOXO3), thereby maintaining the expression of BNIP3 and other mitophagy-related genes. BNIP3 then interacts with and stabilizes PINK1, facilitating the recruitment of PARKIN to mitochondria and activating the PINK1–PARKIN mitophagy pathway [37–41]. In addition, SIRT1 reduces tuberous sclerosis complex 2 (TSC2) acetylation, promoting its translocation to the lysosome. This process inhibits signaling of mechanistic target of rapamycin complex 1 (mTORC1) while activating mitophagy [42]. SIRT1 also indirectly promotes mitophagy by deacetylating and activating the transcription factor EB (TFEB), thereby enhancing its translocation to the nucleus. Once activated, TFEB upregulates the coordinated lysosomal expression and regulation (CLEAR) gene network, leading to increased lysosomal biogenesis and function [43, 44].

SIRT3 also deacetylates FOXO3, promoting mitophagy through the same mechanism as SIRT1 [45, 46]. Furthermore, activation of SIRT3 promotes deacetylation of mitochondrial transcription factor A (TFAM) at K154 residue. Deacetylated TFAM facilitates mitophagy through its interaction with LC3 [47]. Similarly, SIRT3 facilitates the dissociation of hexokinase-2 (HK2) from the voltage-dependent anion-selective channel protein 1 (VDAC1). This dissociation enables the recruitment of PARKIN to VDAC1 [48]. SIRT3 also regulates the acetylation levels of other mitochondrial proteins, such as ATPase inhibitory factor 1 (ATP5IF1), which is essential for PARK2 recruitment and mitophagy [49]. Lastly, SIRT3 helps to maintain a balance between mitochondrial apoptosis and mitophagy. In this sense, SIRT3 suppresses mitochondrial apoptosis by regulating the BCL-2/BAX axis, preventing cytochrome *c* release and apoptosome activation [50].

In contrast to the two previous sirtuins, SIRT2 inhibits mitophagy by promoting microtubule destabilization through the deacetylation of tubulin at K40 residue. This disruption of microtubule stability interferes with both autophagosome formation and its subsequent fusion with lysosomes [51]. Additionally, SIRT2 deacetylates autophagy protein 5 (ATG5), a protein essential for autophagosome elongation. This modification hinders the formation of the ATG5–autophagy-related protein 12 (ATG12)–autophagy-related protein 16-1 (ATG16L1) complex, which is required for the conjugation of LC3B to phosphatidylethanolamine [52].

Meanwhile, SIRT4 regulates mitochondrial dynamics by promoting fusion in an enzymatically dependent manner. It interacts with and stabilizes the long form of optic atrophy protein 1 (L-OPA1), a dynamin-related GTPase, which opposes mitochondrial fission and mitophagy [53].

SIRT5 regulates ammonia-induced mitophagy by modulating glutamine metabolism [54, 55]. It acts through two main mechanisms. First, SIRT5 deacetylates and activates carbamoyl phosphate synthetase 1 (CPS1), the enzyme that catalyzes the first step of the urea cycle, converting ammonia into carbamoyl phosphate for detoxification [56]. Second, it desuccinylates mitochondrial glutaminase (GLS), enhancing the conversion of glutamine to glutamate. The resulting increase in glutamate supports energy production and nitrogen detoxification [57]. Beyond its metabolic functions, SIRT5 modulates PARKIN-dependent mitophagy by inhibiting ULK1 activity through suppression of the PI3K/AKT signaling pathway [58]. Moreover, SIRT5 interacts with pyruvate dehydrogenase kinase isoform 1 (PDK1) and desuccinylates it, thereby inhibiting its activity. This inhibition increases pyruvate oxidation by promoting pyruvate dehydrogenase (PDH) activity, ultimately leading to the suppression of mitophagy [59]. SIRT5 also regulates mitophagy by deacetylating β-tubulin like SIRT2, which reduces microtubule stability [60]. Finally, SIRT5 supports mitochondrial elongation during starvation, protecting against excessive mitophagy. It prevents elevated levels of mitochondrial elongation factor 1 (MIEF1) and FIS1, which otherwise promote OPA1 disassembly and density-regulated protein (DRP1) recruitment and mitochondrial fragmentation [61].

SIRT6 activates AMP-activated protein kinase (AMPK) by increasing the adenosine monophosphate (AMP)/ATP ratio, which in turn stimulates FOXO3 signaling and activates peroxisome proliferator-activated receptor γ coactivator 1α (PGC-1α). FOXO3 signaling enhance the expression and activation of mitophagy-related proteins, facilitating the clearance of damaged mitochondria [62–65]. In addition, AMPK inhibits mTOR signaling by phosphorylating the regulatory-associated protein of mTOR (RAPTOR) [66].

Finally, SIRT7 suppresses mitophagy by maintaining PARKIN in a deacetylated state, thereby inhibiting its activation and mitochondrial recruitment [67].

Another important aspect of sirtuin-mediated regulation of mitophagy is the role of NAD^+^ levels, which directly influence sirtuin enzymatic activity. A metabolic state with an increased [NAD^+^]/[NADH] ratio can affect both the quantity and quality of mitochondria, likely through SIRT1-dependent mitophagy pathways [68]. Mitophagy also plays a key role in limiting excessive stress responses triggered by NAD^+^-consuming enzymes such as PARPs and sirtuins. When mitochondrial quality control fails, these enzymes may become overactivated, leading to uncontrolled NAD^+^ consumption, mitochondrial membrane depolarization, and ultimately, cell death [69].

## Sex differences in sirtuin-mediated mitophagy

Sex-specific differences in sirtuin expression and activity have been observed, largely influenced by sex hormones and age [70]. These sex-related differences may influence how mitophagy is regulated in males and females. However, the precise role of sex in modulating sirtuin-mediated mitophagy remains unclear and requires further investigation. For example, women have higher baseline levels of SIRT1 and SIRT3, as well as mitophagy-related proteins such as BNIP3 and PARKIN, and lysosomal proteins like LAMP1 and cathepsin D, compared to men [71, 72]. These elevated levels are associated with the protective effects mediated by estradiol [71]. After menopause, the decrease in estradiol in women causes a considerable reduction in the levels of SIRT1 and SIRT3, whereas men show a smaller reduction in the levels of these sirtuins during aging [70, 73]. Furthermore, androgens have been shown to down-regulate SIRT6 in the kidney [74]. On the other hand, certain single-nucleotide polymorphisms (SNPs) in the SIRT1 and FOXO3 genes have been associated with a reduced risk of mortality. The protective effect of FOXO3 variants appeared stronger in females, particularly among those who were homozygous for the minor alleles of rs4946936, rs2802292, and rs2253310. These SNPs were linked to decreased mortality risk under both additive and recessive genetic models. In contrast, the protective effect of SIRT1 variants was more pronounced in males. Specifically, male participants homozygous for the minor allele of rs4746720 showed a lower mortality risk under the recessive and heterozygote models [75].

## Sirtuin regulation of mitophagy in physiological conditions and diseases

### Inflammation and injury-related conditions

#### Ischemia–reperfusion (I/R)

I/R phenomenon occurs when tissue suffers from reduced or blocked blood flow, resulting in shortage of oxygen and nutrients. This initial deprivation causes substantial cellular damage. Upon restoration of blood flow, the injury is further exacerbated by a sudden surge in ROS, which inflicts additional harm on cellular components. Moreover, calcium overload and mitochondrial dysfunction activate multiple cell death pathways, compounding the damage. The abrupt return of circulation also triggers a robust inflammatory response, further amplifying tissue injury and contributing significantly to the overall pathology [76].

During myocardial I/R injury, SIRT1 and SIRT3 expression is dysregulated, compromising the heart’s ability to withstand stress [77]. A key consequence of myocardial I/R injury is mitochondrial dysfunction, but this can be mitigated through the activity of checkpoint kinase 1 (CHK1). CHK1 phosphorylates SIRT1 at T530, preventing its degradation by SMAD ubiquitination regulatory factor 2 (SMURF2). Stabilized SIRT1 enhances mitophagy, preserving mitochondrial integrity and reducing myocardial I/R-induced cell damage [78]. Furthermore, the externalization of the phospholipid cardiolipin from the inner mitochondrial membrane to the outer mitochondrial membrane has been proposed as a key signal initiating mitophagy. Once exposed, cardiolipin can serve as a recognition signal for LC3 proteins [79]. In this sense, ginsenoside Rg1 significantly promoted the formation of mitophagosomes, reduced cardiac mitochondrial damage, and enhanced SIRT1/PINK1/PARKIN-mediated mitophagy [80]. In parallel, exosomes enriched with SIRT6 from adipose-derived stem cells also contribute to mitophagy regulation during myocardial I/R injury [81]. Cardiac SIRT6 signaling plays a pivotal role in myocardial protection by initiating a protective cascade that begins with AMPK activation. Activated AMPK subsequently stimulates the FOXO3 pathway, which simultaneously promotes PINK1-PARKIN-mediated mitophagy [63].

Melatonin, when administered at the onset of reoxygenation, also exerts a protective effect by restoring SIRT3 activity, which enhances mitophagy and helps maintain the balance between mitochondrial fission and fusion [82]. Interestingly, melatonin appears to fine-tune mitophagy levels by suppressing apelin expression, thereby downregulating the SIRT3 signaling pathway and preventing excessive mitophagy [83]. Apelin also downregulates SQSTM1 and upregulates LC3B and BECLIN-1 [103]. Additionally, omentin-1, an adipokine primarily secreted by visceral adipose tissue, may activate the SIRT3-FOXO3 axis, further contributing to mitochondrial protection and overall cardiac resilience [84].

On the other hand, in the context of I/R-induced injury to the nervous system, the ceramide kinase-like protein (CERKL) has been shown to play a protective role by promoting mitophagy. Overexpression of CERKL enhances the stability of SIRT1, which in turn upregulates the expression of key mitophagy-related proteins PINK1 and PARKIN in neuronal cells [85]. In contrast, the long non-coding RNA taurine upregulated gene 1 (TUG1) exerts an opposing effect by suppressing SIRT1 expression. TUG1 promotes SIRT1 degradation through enhanced ubiquitination mediated by the E3 ligase F-box/WD repeat-containing protein 7 (FBXW7) [86]. In response to ischemic stroke, neurons initiate a protective mechanism involving ubl carboxyl-terminal hydrolase 18 (USP18), which stabilizes the fat mass and obesity-associated protein (FTO) through deubiquitination, leading to increased FTO protein levels. Elevated FTO reduces N^6^-methyladenosine (m6A) modification on SIRT6 mRNA in a manner dependent on YTH domain-containing family protein 2 (YTHDF2). This reduction in m6A tagging prevents mRNA degradation, thereby enhancing SIRT6 expression. Upregulated SIRT6 increases mitophagy-related proteins, supporting mitochondrial quality control and reducing neuronal damage caused by ischemic stroke [62].

The annexin-A1 small peptide (ANXA1sp) protects against kidney injury by upregulating SIRT3 expression and promoting mitophagy [87].

Lastly, in the context of lung I/R injury under diabetic conditions, adiponectin plays a vital protective role by mitigating oxidative stress, inflammation, apoptosis, and mitochondrial dysfunction. This protective effect is primarily mediated through the activation of mitophagy via the AMPK-SIRT1-PINK1 signaling pathway. By stimulating this pathway, adiponectin promotes the selective clearance of damaged mitochondria, which in turn supports the restoration of mitochondrial function and cellular homeostasis [88].

#### Sepsis-induced organ injury

Sepsis is a life-threatening condition that arises from a dysregulated and excessive immune response to infection. Rather than effectively containing the invading pathogen, the immune system’s overactivation leads to systemic inflammation, widespread tissue damage, and ultimately, the failure of multiple organs and physiological systems [89].

Emerging evidence highlights the relevant role of mitophagy in protecting cardiac function during sepsis-induced myocardial injury. A key regulator in this process is the basic helix-loop-helix ARNT-like protein 1 (BMAL1), which enhances mitophagy by activating SIRT1. This activation is mediated through BMAL1-driven transcription of nicotinamide phosphoribosyltransferase (NAMPT), leading to elevated NAD + levels and subsequent SIRT1 stimulation [90]. In parallel, ANXA1sp has also been shown to support mitochondrial quality control in septic hearts by upregulating SIRT3 expression and promoting mitophagy [91].

A similar SIRT3-mediated protective mechanism has been observed in the context of sepsis-induced acute lung injury (ALI) [92]. In addition to SIRT3, SIRT1 interacts with the small GTPase RAB7 in late endosomes, ensuring the timely removal of damaged mitochondria. This process is crucial to prevent overactivation of inflammatory pathways such as stimulator of interferon genes (STING) and NOD-like receptor protein 3 (NLRP3) inflammasome [93]. In the same direction, MAP kinase kinase 3 (MAPKK3) deficiency contributes to greater cellular resilience and significantly lowers sepsis-related mortality in mice, in part by reducing lung injury and preserving tissue function. MAPKK3 deficiency enhances both mitochondrial biogenesis and mitophagy by upregulating key regulators such as SIRT1, PGC-1α, and nuclear respiratory factor 1 (NRF1) [94].

In sepsis-induced acute kidney injury, bone marrow-derived mesenchymal stem cells (BMSCs) suppressed inflammation, apoptosis, and pyroptosis, while promoting mitophagy in renal tubular epithelial cells. These effects were associated with the upregulation of PARKIN and SIRT1 expression [95]. In particular, zinc supplementation increases PARKIN acetylation through inhibition of SIRT7 activity, leading to increased mitophagy and suppression of NLRP3 inflammasome activation and pyroptosis. This protective mechanism helps alleviate sepsis-induced acute kidney injury [67].

#### Liver fibrosis

Liver fibrosis is characterized by the excessive accumulation of extracellular matrix proteins and results from a maladaptive wound healing response to chronic liver injury and inflammation. This progressive condition can eventually lead to cirrhosis and liver failure. Major causes of liver fibrosis include nonalcoholic fatty liver disease (NAFLD), chronic viral hepatitis, alcoholic liver disease, and cholestatic liver disorders [96]. In models of liver injury induced by a high-fat diet, SIRT3 expression is down-regulated, leading to decreased mitophagy. This alteration contributes to mitochondria-dependent hepatocyte apoptosis and liver tissue damage [97]. Furthermore, SIRT3 modulates the acetylation of the non-neuronal SNAP25-like protein 1 (NIPSNAP1), a mitophagy adaptor protein. This action is important for maintaining mitochondrial quality control during the progression of liver fibrosis [98].

#### Neuroinflammation

Neuroinflammation is an immune response within the central nervous system (CNS) and represents a significant threat to human health [99]. In this regard, when mitophagy is disrupted, damaged mitochondria accumulate, leading to increased production of ROS and the release of pro-inflammatory cytokines. This cascade not only promotes neuroinflammation but also contributes to progressive neuronal degeneration [100]. Dexmedetomidine treatment has been shown to effectively reduce neuroinflammation and improve cognitive function by upregulating SIRT3, which in turn enhances SIRT3-mediated mitophagy [101]. Similarly, SIRT1 plays a neuroprotective role by promoting mitophagy through the deacetylation of FOXO1. This mechanism is particularly important in microglia, where elevated SIRT1 levels help mitigate neuroinflammation following subarachnoid hemorrhage [102]. In contrast to the beneficial roles of SIRT1 and SIRT3, SIRT2 appears to have an opposing effect. Upon translocation to the nucleus, SIRT2 inhibits mitophagy, thereby potentially exacerbating neuroinflammation. However, this detrimental pathway can be counteracted by miR-486-3p, which targets SIRT2. By downregulating SIRT2, miR-486-3p promotes mitophagy and facilitates a shift in microglial phenotype from the pro-inflammatory M1 state to the anti-inflammatory M2 state, ultimately reducing neuroinflammation in subarachnoid hemorrhage [103].

### Neurological and neurodegenerative disorders

#### Alzheimer’s disease

Alzheimer’s disease (AD) is the most common form of dementia. Most cases (> 95%) are sporadic, characterized by a late onset between 80 and 90 years of age, and are mainly due to impaired clearance of amyloid-β peptides (Aβ) from the brain. In contrast, less than 1% of cases are familial, caused by inherited mutations that alter Aβ processing, with an average onset around 45 years of age [104]. Animal models, such as APP/PS1 and ApoE4 mice, have provided a better understanding of the role of sirtuins in this disease [105]. APP/PS1 mice co-express human mutant amyloid precursor protein (APP) and presenilin-1 (PS1), leading to increased production of Aβ (particularly Aβ42), early plaque formation, neuroinflammation, and cognitive deficits, making them a robust model for amyloid pathology. In contrast, ApoE4 mice express the human apolipoprotein E4 (APOE4) allele, the strongest genetic risk factor for late-onset Alzheimer’s disease [106]. In the hippocampus of APP/PS1 mice, levels of NAMPT and NAD^+^ are reduced, leading to decreased activity of SIRT1 and SIRT3 [107]. Apigenin helps preserve the NAD^+^/NADH ratio to maintain sirtuin activity. By enhancing SIRT3 function, apigenin promotes proper mitophagy. This mitochondrial quality control mechanism contributes to neuronal protection and helps mitigate disease progression [108]. Similarly, ApoE4 mice exhibit reduced SIRT3 and FOXO3 expression, resulting in impaired mitophagy [109]. Pharmacological interventions targeting these pathways restore mitophagy and improve cognitive function [107]. In particular, honokiol, a natural lignan, enhances learning and memory in APP/PS1 mice by activating SIRT3-mediated mitophagy [110]. Likewise, aloe-emodin activates mitophagy via the AMPK/PGC-1α/SIRT3 pathway, protecting hippocampal neurons and helping alleviate cognitive dysfunction in Alzheimer’s disease [111]. Exercise similarly restores PINK1/PARKIN-mediated mitophagy in the hippocampus of APP/PS1 mice by activating the SIRT1-FOXO1/3 axis [41]. A comparable effect was observed in a zebrafish model, where swimming exercise increased mitophagosome formation and SIRT1 activation [112]. In this regard, the ketone body β-hydroxybutyrate produced after endurance exercise promotes nuclear localization of FOXO1, FOXO3a, and PGC-1α in a SIRT2 silencing-dependent manner. This activation enhances pathways leading to both mitophagy and mitochondrial biogenesis, thereby increasing neuronal resilience and supporting cell survival and function under metabolically challenging conditions [113].

#### Hearing dysfunction disorders

In hearing dysfunction disorders, mitochondrial quality control has emerged as a key factor in the progression of auditory damage. Patients with OPA1 mutations often exhibit a clinical profile consistent with auditory neuropathy, characterized by moderate hearing loss, disproportionately impaired speech perception, abnormal auditory brainstem responses, and preserved otoacoustic emissions [114]. Animal studies using the OPA1 delTTAG allele mouse model, which mimics the human condition of dominant optic atrophy, have shown an early increase in SIRT3-mediated mitophagy. While initially protective, this upregulation ultimately leads to excessive degradation of mitochondria and triggers apoptosis of inner hair cells, contributing to hearing impairment [115]. Additionally, miR-34a has been identified as a key regulator of mitochondrial homeostasis in auditory cells. By directly targeting and suppressing SIRT1, miR-34a inhibits both mitophagy and mitochondrial biogenesis. This suppression increases susceptibility to oxidative stress and promotes cell death in organ of Corti cells, which are essential for converting sound vibrations into neural signals [116].

#### DNA repair syndromes

Sirtuins play a fundamental role in DNA repair [117]. Ataxia-telangiectasia (A-T) is a neurodegenerative disorder caused by mutations in the ATM gene, leading to impaired DNA repair, progressive neurodegeneration, and immune dysfunction. Treatments that boost intracellular NAD^+^ levels, such as nicotinamide riboside (NR), SRT1720, and olaparib, enhance mitophagy in ATM-deficient neurons. These interventions help to mitigate A-T-related neuropathology by restoring neuromuscular function, slowing memory decline, and extending lifespan in animal models [118]. In the context of DNA damage induced by irradiation, hematopoietic stem cells (HSCs) activate protective pathways to maintain function. Treatment with 16,16-dimethyl prostaglandin E2 (dmPGE2) increases SIRT1 expression and activity via the PKA pathway. This activation is associated with epigenetic suppression of TP53 and enhanced mitophagy, supporting stem cell maintenance under genotoxic stress [119]. On the other hand, Xeroderma pigmentosum group A (XPA) is a rare genetic disorder characterized by extreme sensitivity to ultraviolet (UV) light due to defects in nucleotide excision repair (NER). XPA is also associated with defective mitophagy, marked by excessive PINK1 cleavage and increased mitochondrial membrane potential. These abnormalities appear to result from reduced activation of the NAD^+^–SIRT1–PGC-1α axis, which is suppressed by hyperactivation of the DNA damage sensor poly(ADP-ribose) polymerase 1 (PARP1). Notably, this phenotype can be reversed through PARP1 inhibition or supplementation with NAD^+^ precursors [120].

### Cardiometabolic conditions

#### Cardiovascular pathologies

During hypertension, SIRT4 acts as a negative regulator of SIRT3, thereby suppressing SIRT3-mediated mitophagy and inhibiting the deacetylation of manganese superoxide dismutase (MnSOD) at K122 residue. This inhibition leads to an increase in mitochondrial ROS accumulation which, if chronically maintained, contributes to the onset of cellular senescence [121]. In contrast, SIRT3 overexpression promotes PINK1/PARKIN-mediated mitophagy in cardiac microvascular endothelial cells, leading to a reduction in mitochondrial ROS production and the enhancement of angiogenic processes. This restoration of mitochondrial homeostasis facilitates vascular sprouting and tube formation [122]. Similarly, elevating NAD^+^ levels or overexpressing SIRT1 activates key protective pathways, including SIRT1-PINK1 and SIRT1-GPX4 signaling. These pathways enhance mitophagy while suppressing ferroptosis, thus contributing to improved mitochondrial function [123].

Mitochondrial isocitrate dehydrogenase (IDH2) deficiency in endothelial cells activates miRNA miR-34b/c by reducing α-ketoglutarate (α-KG) levels. miR-34b/c targets the 3′ untranslated region of SIRT3 mRNA, disrupting mitophagy and increasing mitochondrial ROS, ultimately leading to endothelial senescence [124]. Supplementation with α-KG attenuates endothelial senescence and myocardial hypertrophy, fibrosis, and chronic heart failure induced by angiotensin II (AngII)-mediated pressure overload [123]. Quercetin enhances SIRT1-mediated mitophagy in renal tubular epithelial cells exposed to AngII, exerting a protective antifibrotic effect [125]. Likewise, dapagliflozin (DAPA), a reversible inhibitor of sodium-glucose cotransporter 2 (SLC5A2) in the renal proximal convoluted tubule, can reduce cardiac fibrosis and atrial arrhythmias by enhancing SIRT1-mediated mitophagy. Mechanistically, DAPA treatment decreases SIRT1 phosphorylation, a modification necessary for its ubiquitination and degradation, thereby stabilizing and activating SIRT1 [126]. Melatonin inhibited atherosclerotic progression, at least in part, by promoting SIRT3-mediated mitophagy [127].

In the setting of viral myocarditis, semaphorin-3A (SEMA3A), a secreted signaling molecule involved in cell migration, growth, and survival, plays a protective role in cardiomyocyte maintenance. It promotes mitophagy and inhibits inflammasome activation via the positive regulation of SIRT1, acting through intracellular signaling cascades such as the PI3K/AKT pathway [128].

#### Diabetes

Diabetes is a condition characterized by elevated blood glucose levels, resulting from insufficient insulin production by the pancreas or impaired cellular response to insulin. Sirtuins play a crucial role in diabetes by regulating key metabolic processes, including hepatic glucose homeostasis, pancreatic β-cell insulin secretion, skeletal muscle metabolic balance, adipocyte energy regulation, and insulin sensitization, among others. Overall, sirtuins are pivotal for optimizing blood glucose levels in diabetic patients [129].

Hyperglycemia downregulates SIRT3 transcription and enhances its degradation, promoting metabolic reprogramming and inflammatory activation, particularly in diabetic neuropathic pain [130]. In more detail, elevated glucose levels activate the AKT pathway in microglia, leading to inactivation of FOXO1 and further suppression of SIRT3 transcription [131]. Overexpression of SIRT3 alleviates this condition by activating the FOXO3A-PINK1-PARKIN-mediated mitophagy pathway, improving mitochondrial quality control and reducing cellular stress [130].

Similarly, in diabetic cardiomyopathy, downregulation of SIRT3 impairs mitophagy [132]. Serine/threonine-protein kinase 4 (STK4) exacerbates this process by suppressing SIRT3 expression, resulting in mitochondrial dysfunction, oxidative stress, and impaired cellular function [133].

Beyond neuropathy and cardiomyopathy, SIRT3 also plays a critical role in other diabetic complications. It promotes corneal epithelial wound healing [134] and protects retinal pigment epithelial cells from high glucose-induced apoptosis by regulating mitophagy through the FOXO3A-PINK1-PARKIN pathway [135]. Additionally, SIRT3 activates the AMPK/mTOR/ULK1 signaling cascade, where AMPK activation enhances SIRT3 and ULK1 activity while inhibiting mTOR, further supporting mitochondrial health under hyperglycemic conditions [136].

SIRT5 also plays a significant metabolic role by stabilizing uncoupling protein 1 (UCP1) through desuccinylation at K56 and K151 residues. SIRT5 regulates the succinylation and malonylation of multiple mitochondrial proteins, maintaining mitochondrial metabolic flexibility. In the absence of SIRT5, excessive succinylation and malonylation disrupt mitophagy, impairing the tissue’s ability to shift between glucose and fat metabolism, and resulting in altered responses to cold stress in brown adipose tissue [137].

Finally, the BMAL1/SIRT1/PGC-1α mitophagy pathway appears to protect podocytes, specialized cells of the Bowman’s capsule that encase the glomerular capillaries, under high glucose conditions, a major contributor to the pathogenesis of diabetic nephropathy. Moreover, activation of the SIRT1-PGC-1α-TFAM pathway in placenta-derived mesenchymal stem cells restores PINK1/PARKIN-mediated mitophagy following podocyte injury, thereby helping to slow the progression of diabetic nephropathy [138].

### Aging-related disorders

#### Reproductive aging

Reproductive aging in female mammals is an irreversible process primarily driven by the progressive decline in oocyte quality, which represents the key limiting factor in fertility. A growing body of evidence indicates that mitochondrial dysfunction plays a central role in ovarian aging [139]. As oocytes age, levels of NAD^+^ also decline, further impairing mitochondrial and sirtuin function and overall cellular metabolism. Therapeutic strategies aimed at restoring NAD^+^ levels have shown promise in reversing aspects of reproductive aging [140]. Melatonin utilizes SIRT3-mediated mitophagy to reduce oxidative stress-induced apoptosis and mitochondrial damage in ovarian granulosa cells, thereby preserving cell function and viability [40]. Additionally, melatonin acts through adipose-derived stem cells to trigger the SIRT6/NF-κB pathway in ovaries. This pathway not only promotes mitophagy but also regulates mitochondrial fission and enhances mitochondrial biogenesis, further supporting mitochondrial health and ovarian longevity [141]. Similarly, the natural compound nobiletin, a polymethoxylated flavonoid, has been shown to delay ovarian aging by activating SIRT1-mediated mitophagy, thereby maintaining oocyte quality [142]. Furthermore, SIRT2 plays a regulatory role in cumulus cell apoptosis, likely through its influence on mitophagy mediated by mitogen-activated protein kinase 15 (MAPK15) [143].

#### Osteoarthritis

Osteoarthritis (OA) is the most prevalent joint disorder, primarily affecting diarthrodial joints [144]. Emerging evidence highlights the critical role of mitochondrial quality control, particularly mitophagy, in maintaining joint health and slowing OA progression [145, 146]. One key regulator is 17β-estradiol (17β-E2), which enhances SIRT1 expression and activates the AMPK signaling pathway while suppressing mTOR activity. This coordinated signaling promotes the formation of mitochondrial autophagosomes, enhancing mitophagy in chondrocytes. By facilitating the clearance of damaged mitochondria, this mechanism preserves mitochondrial integrity and supports chondrocyte viability. The decline in 17β-E2 levels following menopause may impair this protective pathway, contributing to OA onset and progression [147]. In this context, resveratrol treatment protected osteoblasts by promoting SIRT1-mediated mitophagy and thereby supporting bone health in OA conditions [148]. Similarly, honokiol can reverse mitophagy impairments caused by hyperglycemia. It restores SIRT3 expression and activity, promoting mitophagy and thereby decelerating OA progression [149]. Other molecules also influence mitophagy via SIRT3 signaling. Growth/differentiation factor 11 (GDF11) enhances osteogenic differentiation of stem cells through the SIRT3–FOXO3 pathway [45], while irisin, a myokine, activates SIRT3 through the AMPK–PGC-1α axis in adipose-derived stem cells. This action helps preserve cell viability and osteogenic potential, particularly after injury induced by advanced glycation end products (AGEs) [150]. In contrast, interleukin-1β (IL-1β) promotes chondrocyte apoptosis by downregulating SIRT3 expression and impairing mitophagy. However, treatment with mitochonic acid-5 (MA-5) counteracts these effects, thereby protecting cartilage cells [151].

On the other hand, not all SIRT3-related mechanisms are beneficial, transfer RNA (tRNA) (guanine-N(7)-)-methyltransferase (METTL1)-mediated N^7^-methylguanosine (m7G) modification increases levels of a mitochondrial RNA-derived fragment, mt-tRF3b-LeuTAA, which downregulates sentrin-specific protease 1 (SENP1) and enhances SUMOylation of SIRT3. This post-translational modification boosts SIRT3 deacetylase activity. While moderate SIRT3 activation supports cellular health, excessive SIRT3 activity in OA has been linked to chondrocyte degeneration and accelerated cartilage degradation [152].

#### Intervertebral disc degeneration

Human intervertebral discs experience degenerative changes with age, which are major contributors to common impairments and disabilities in middle-aged and older adults [153]. Lumbar disc degeneration is a leading cause of chronic low back pain. It typically begins with an imbalance between catabolic and anabolic processes in the intervertebral discs, resulting in extracellular matrix degradation. This degradation promotes neoinnervation and neovascularization, leading to loss of water content, disc bulging, nucleus pulposus degeneration, and reduced disc height [154]. circERCC2 and circSPG21 regulate SIRT1 expression in degenerative nucleus pulposus tissues by negatively controlling miR-182-5p and miR-217, respectively. Down-regulation of these miRNAs results in increased levels of SIRT1, which promotes beneficial mitophagy that prevents apoptosis of nucleus pulposus cells and contributes to the attenuation of intervertebral disc degeneration [155, 156]. Furthermore, SIRT1 mitigates IL-1β-induced NLRP3 inflammasome activation in nucleus pulposus cells during intervertebral disc degeneration by promoting mitophagy [157].

### Cancer

In the context of cancer, mitophagy plays a complex and dualistic role, acting as both a survival mechanism and a potential vulnerability. On one hand, mitophagy can support tumor cell survival by removing damaged mitochondria, thereby reducing oxidative stress and maintaining mitochondrial function. This allows cancer cells to adapt to the metabolic stress induced by chemotherapy and radiotherapy, ultimately enhancing their resistance to treatment [158]. In this sense, SIRT1-mediated mitophagy is involved in the hormonal resistance of endometrial cancer [38]. However, cyclin-dependent kinase 9 (CDK9) can inhibit this mitophagy process by phosphorylating SIRT1 at S47, which suppresses its deacetylase activity [37]. Similarly, oleanolic acid activates pro-survival mitophagy in colon cancer cells through the MAPK11/FOXO3a/SIRT6 signaling pathway [64].

On the other hand, impaired mitophagy leads to the accumulation of dysfunctional mitochondria, resulting in insufficient ATP production and elevated levels of ROS, which can trigger cancer cell death. Conversely, excessive mitophagy may also be detrimental to tumor cells. By eliminating too many mitochondria, even functional ones, the process can compromise cellular energy homeostasis and induce apoptosis, making mitophagy a potential target for therapeutic exploitation [159]. In this context, vincristine induces mitophagy in breast cancer cells by disrupting the interaction between heat shock 70 kDa protein 9 (HSP70) and SIRT2. This disruption leads to increased acetylation of HSP70 at K126, enhancing its ability to bind and sequester BCL-2. The HSP70–BCL-2 complex facilitates the formation of mitophagosomes, thereby promoting mitophagy and contributing to vincristine’s anticancer activity [160]. In a similar way, excessive mitophagy driven by the SIRT3-FOXO3A-PINK1-PARKIN pathway leads to a reduction in RING finger protein 1B (RING1b) expression. This decrease in RING1b impairs the ubiquitination of histone H2A at K119, a modification crucial for chromatin remodeling. As a result, the relaxed chromatin state facilitates more efficient cancer DNA damage following radiation exposure [161]. Tetrandrine, an anticancer compound extracted from *Stephania tetrandra*, promotes the degradation of SIRT5 through the ubiquitin–26S proteasome pathway, leading to a significant reduction in its levels. This decrease in SIRT5 impairs mitochondrial function and results in the accumulation of ROS and excessive mitophagy [162].

Newcastle disease virus (NDV), a member of the Paramyxoviridae family, is currently being explored as a promising oncolytic agent for cancer therapy. Upon infection, NDV induces mitochondrial dysfunction characterized by structural damage, elevated mitochondrial ROS, and impaired electron transport chain activity. This mitochondrial stress activates the AMPK–mTOR signaling axis, which in turn promotes the lysosomal degradation of SIRT3. The loss of SIRT3 leads to the stabilization of hypoxia-inducible factor 1α (HIF1A) and triggers excessive autophagy driven by aberrant mTOR signaling crosstalk, ultimately contributing to NDV’s oncolytic activity [163].

### Response to pollutants and toxicants

Environmental pollutants are compounds that have been released into the ecosystem and can pose a serious threat to the well-being of living beings [164]. Since these compounds are in principle ubiquitous and many of them quite resistant to degradation, we are exposed to numerous on a daily basis [165].

In this regard, burnt tobacco smoke contains over 3,800 chemicals, more than half of which are classified as potential toxins or carcinogens [165]. In mice exposed to cigarette smoke, SIRT1 deficiency exacerbated airway resistance and cellular senescence, accompanied by increased acetylation of FOXO3, reduced PINK1 protein levels, and impaired mitophagy [39].

Sodium arsenite, a compound with known carcinogenic and teratogenic properties, is primarily used as a pesticide but also serves in hide preservation, antiseptics, and as an additive in dyeing and soap production. Exposure to this chemical also suppresses SIRT1 expression, whereas resveratrol-mediated activation of SIRT1 mitigates sodium arsenite-induced acute kidney injury by enhancing mitophagy [166].

Rotenone, approved as a piscicide and formerly used as a broad-spectrum insecticide, disrupts mitochondrial function by inhibiting complex I of the electron transport chain. Ghrelin counteracts rotenone-induced cytotoxicity by activating the AMPK/SIRT1/PGC-1α signaling pathway, which enhances mitophagy, facilitating the removal of damaged mitochondria and reducing cellular stress [167].

Deoxynivalenol (DON), a mycotoxin predominantly found in grains such as wheat, barley, oats, rye, and corn, is primarily associated with *Fusarium graminearum* contamination. DON exposure reduces tryptophan uptake in the brain, impairing serotonin synthesis and contributing to its anorexic effects. Additionally, DON inhibits mitophagy by promoting the dephosphorylation and inactivation of SIRT1 [168].

## Pharmacological implications

Many polyphenols, including those currently being evaluated in clinical trials, such as resveratrol, quercetin, and curcumin, have been shown to provide health benefits by influencing mitophagy, primarily through their activation of SIRT1 [125, 148, 169]. Other natural compounds with proven health benefits, such as honokiol and tetrandrine, have similar effects [110, 162]. Melatonin is another well-studied natural molecule in this context, with consistent evidence supporting its ability to beneficially enhance mitophagy in various physiological and pathological conditions [47, 82, 127, 170, 171]. Indeed, melatonin is currently the only compound being investigated in clinical trials specifically targeting mitophagy. A trial (NCT03897101) is evaluating whether melatonin-induced mitophagy can help reduce inflammation and alleviate metabolic acidosis in newborns.

While synthetic inhibitors and activators have been developed for several sirtuins, their clinical translation remains limited. Notably, SIRT4 remains an outlier, as no specific synthetic modulators have yet been identified, reflecting a need for further exploration. In particular, SIRT1 has numerous specific inhibitors identified. Among them, selisistat (EX-527) is the only one that has progressed to clinical trials [172]. Furthermore, some SIRT1 activators, including compounds like DCHC, have demonstrated beneficial effects in models of mitochondrial damage [173]. SIRT2 also has several commercial inhibitors, such as thiomyrristoyl, AC-93253 and AK-7, which are mainly used in cancer research [174]. However, none of these have advanced beyond laboratory studies. In the case of SIRT3, the activator SKLB-11A has demonstrated the ability to stimulate autophagy-related signaling and mitigate mitochondrial dysfunction, particularly in models of doxorubicin-induced cardiotoxicity [175]. This suggests a potential link between sirtuin modulation and the regulation of mitophagy. SIRT5 inhibitors such as Et-29 and sirtuin-IN-2 have been investigated for their potential to modulate cancer metabolism, showing promising effects in in vitro models [176, 177]. In the case of SIRT6, the activator UBCS039 has been shown to stimulate autophagy pathways [178]. Conversely, SIRT6 inhibitors, including SIRT6-IN-3 and SIRT6-IN-4, have been reported to induce apoptosis in cancer cell lines [179]. As for SIRT7, two inhibitors, 97,491 and YZL-51N, have been identified, but their use has so far been limited to in vitro studies, and their biological effects remain to be validated [180, 181]. These findings point to the need for further research to assess the therapeutic relevance of these compounds and to advance them beyond early-stage cellular models.

## Conclusions and future perspectives

Mitophagy is a vital cellular process whose effects can be either protective or harmful. On the beneficial side, it helps maintain mitochondrial health by selectively removing damaged mitochondria, thereby reducing oxidative stress and preserving optimal cellular function. However, when overactivated, mitophagy can become detrimental by eliminating not only defective but also healthy mitochondria, which may disrupt energy production and trigger apoptosis. Therefore, tight regulation of mitophagy is essential to ensure cellular homeostasis and survival.

**Fig. 4:**
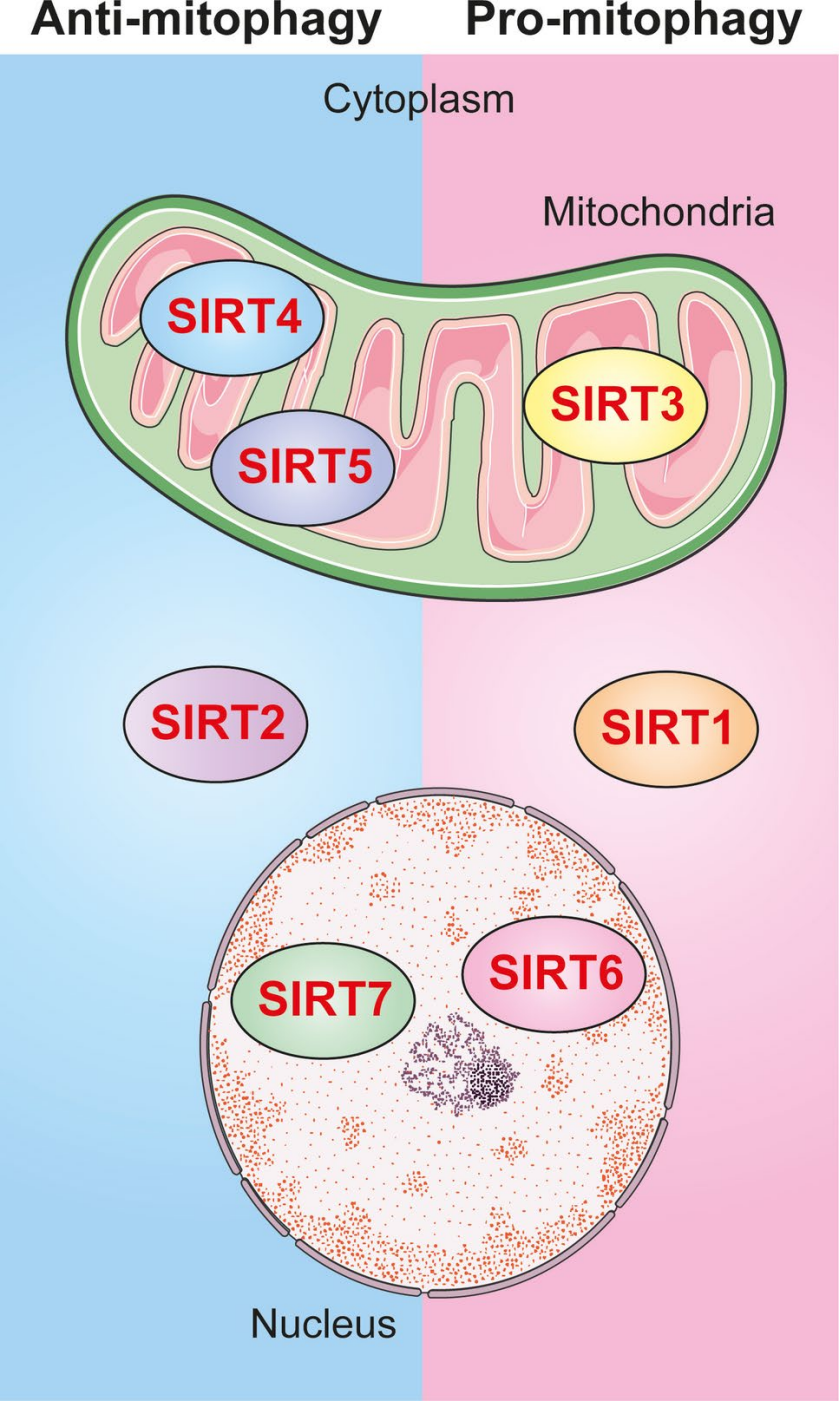
Differential roles of sirtuins in mitophagy. This figure illustrates the distinct roles of sirtuin family members in the regulation of mitophagy. SIRT1, SIRT3, and SIRT6 generally act as positive regulators, promoting mitophagy, while SIRT2, SIRT4, SIRT5, and SIRT7 are more commonly associated with inhibitory or limiting effects on the process. The subcellular localization of each sirtuin is also depicted, highlighting their compartment-specific functions

Sirtuins are key regulators of mitophagy and their influence on this process varies according to the cellular context and environmental conditions (Fig. 4). Overall, SIRT1, SIRT3, and SIRT6 act to promote mitophagy, whereas SIRT2, SIRT4, SIRT5, and SIRT7 tend to inhibit or limit it. SIRT1 and SIRT3 are the best characterized members of the sirtuin family in the context of mitophagy. SIRT1 mainly facilitates mitophagy through activation of the transcription factors FOXO1 and FOXO3, which up-regulate genes involved in mitochondrial degradation, whereas SIRT3 promotes mitophagy solely through activation of FOXO3. SIRT6 indirectly participates in mitophagy through stimulation of AMPK, an energy sensor that subsequently increases FOXO3 activity. Among the negative regulators of mitophagy, SIRT2 impairs the process by interfering with the formation and elongation of autophagosomes to engulf and degrade damaged mitochondria. SIRT4 disrupts mitochondrial dynamics that are critical for labeling dysfunctional mitochondria and initiating mitophagy. SIRT5 supports metabolic homeostasis by regulating key metabolic enzymes, thereby reducing mitochondrial stress and decreasing the cellular need to activate mitophagy. SIRT7 suppresses mitophagy more directly through deacetylation of PARKIN.

It is important to note that NAD^+^, the main regulator of sirtuin activity, also plays a beneficial role in promoting mitophagy when its levels are elevated in a controlled and transient manner. Increased availability of NAD^+^ potentiates the activity of SIRT1 and SIRT3, which in turn stimulate the removal of damaged mitochondria, thereby promoting cellular health and mitochondrial quality control. However, chronic or excessive elevation of NAD^+^ (e.g., starvation) can have detrimental effects. This is because overactivation of NAD^+^-dependent enzymes, particularly sirtuins and poly(ADP-ribose) polymerases (PARPs), can lead to cellular stress, altered energy metabolism, and ultimately cell death.

Sirtuin-mediated regulation of mitophagy plays a crucial role in a wide range of physiological and pathological processes. Physiologically, it is especially relevant in delaying reproductive aging and in the body’s response to environmental pollutants and toxicants. In pathological contexts, increased mitophagy, when properly regulated, has been shown to be beneficial in conditions such as ischemia–reperfusion (I/R) injury in various tissues, sepsis-induced organ damage, neuroinflammation, osteoarthritis, intervertebral disc degeneration, Alzheimer’s disease, DNA repair disorders, and cardiovascular diseases such as hypertension, atherosclerosis, and viral myocarditis, as well as liver fibrosis. Conversely, in certain conditions such as hearing loss disorders, excessive mitophagy can have detrimental effects. In the context of cancer, mitophagy has a dual role, as it promotes the survival of cancer cells under stress and, at the same time, represents a potential therapeutic vulnerability.

As discussed throughout the manuscript, various substances, including natural compounds, pharmaceuticals, environmental pollutants, and toxic agents, can influence mitophagy by modulating the activity of sirtuins. However, isoform-specific activators or inhibitors are lacking for most sirtuins. Furthermore, the pharmacokinetics and pharmacodynamics of existing sirtuin modulators remain poorly understood, so research in this field is highly commendable. Similarly, lifestyle factors, such as diet and exercise, play an important role. Diets rich in sugars and fats may impair mitophagy by suppressing the activity of certain sirtuins, whereas regular physical activity may enhance mitophagy by promoting favorable cellular and metabolic conditions.

Despite significant advances in understanding how sirtuins regulate mitophagy, many relevant questions remain unanswered. Much of the current knowledge focuses on SIRT1 and SIRT3, whereas the functions of other isoforms (SIRT2, SIRT4, SIRT5, SIRT6, and SIRT7) have yet to be fully elucidated. Future research should aim to further uncover the specific molecular mechanisms by which these other sirtuins influence mitophagy. Furthermore, sirtuins appear to exert tissue-specific effects, and the regulation of mitophagy by different sirtuin isoforms may vary across tissues and age groups, warranting further investigation. Evaluating tissue-specific mitophagic activity and its association with sirtuin expression and function may provide a valuable foundation for further investigations in this field. Equally important is to identify the cellular and environmental conditions that selectively activate each sirtuin isoform and to determine how their activities are coordinated during the mitophagic process. In this context, lifestyle interventions, such as changes in diet and physical activity, can help to begin to address this knowledge gap. Comparison of mitophagic activity and its correlation with sirtuin expression and activity between sedentary and physically active individuals, as well as between those following a high-fat diet and those following a Mediterranean diet (known for its health benefits), could provide valuable information. Given that sirtuin function is closely tied to subcellular localization, understanding how their compartmentalization affects mitophagy regulation is also essential. Taking advantage of the omics data currently available, some research groups may try to unravel the inter-organelle communication pathways that are critically involved in the regulation of mitophagy.

From a translational perspective, there is growing interest in developing therapeutic strategies that modulate mitophagy via sirtuins. Such interventions could potentially delay aging, enhance healthspan, and offer preventive or therapeutic benefits for various diseases. Ideally, these strategies would incorporate personalized approaches combining diet, physical activity, and targeted supplementation. However, achieving this remains a complex challenge that requires a lot of work and effort. Additionally, it is worth exploring whether the sirtuin-mitophagy axis could serve as a diagnostic or prognostic biomarker for specific health conditions, opening new avenues for early detection and intervention.

## Data Availability

No datasets were generated or analysed during the current study.
